# Characterization of physicochemical properties of ivy nanoparticles for cosmetic application

**DOI:** 10.1186/1477-3155-11-3

**Published:** 2013-02-01

**Authors:** Yujian Huang, Scott C Lenaghan, Lijin Xia, Jason N Burris, C Neal Jr Stewart, Mingjun Zhang

**Affiliations:** 1 Department of Mechanical, Aerospace and Biomedical Engineering, University of Tennessee, Knoxville, TN, 37996, USA; 2 Department of Plant Sciences, University of Tennessee, Knoxville, TN, 37996, USA

**Keywords:** Ivy nanoparticle, UV extinction, Sunscreen, Physicochemical property

## Abstract

**Background:**

Naturally occurring nanoparticles isolated from English ivy (*Hedera helix*) have previously been proposed as an alternative to metallic nanoparticles as sunscreen fillers due to their effective UV extinction property, low toxicity and potential biodegradability.

**Methods:**

This study focused on analyzing the physicochemical properties of the ivy nanoparticles, specifically, those parameters which are crucial for use as sunscreen fillers, such as pH, temperature, and UV irradiation. The visual transparency and cytotoxicity of ivy nanoparticles were also investigated comparing them with other metal oxide nanoparticles.

**Results:**

Results from this study demonstrated that, after treatment at 100°C, there was a clear increase in the UV extinction spectra of the ivy nanoparticles caused by the partial decomposition. In addition, the UVA extinction spectra of the ivy nanoparticles gradually reduced slightly with the decrease of pH values in solvents. Prolonged UV irradiation indicated that the influence of UV light on the stability of the ivy nanoparticle was limited and time-independent. Compared to TiO_2_ and ZnO nanoparticles, ivy nanoparticles showed better visual transparency. Methylthiazol tetrazolium assay demonstrated that ivy nanoparticles exhibited lower cytotoxicity than the other two types of nanoparticles. Results also suggested that protein played an important role in modulating the three-dimensional structure of the ivy nanoparticles.

**Conclusions:**

Based on the results from this study it can be concluded that the ivy nanoparticles are able to maintain their UV protective capability at wide range of temperature and pH values, further demonstrating their potential as an alternative to replace currently available metal oxide nanoparticles in sunscreen applications.

## Background

Titanium dioxide (TiO_2_) and zinc oxide (ZnO) nanoparticles have been widely used as commercial sunscreen fillers due to their ability to absorb and scatter UV light [1-3]. TiO_2_ crystals absorb UVB radiation from 280 to 315 nm, while ZnO crystals absorb UVA radiation from 315 to 400 nm; therefore the combined use of both particles provides the UV protection in a broad spectra [4]. Later, silicon (Si) nanoparticles were also proposed to achieve the same purpose [5]. The advantage of using nanoparticles, as opposed to micro-sized particles, is the transparency of nanoparticles to visible light, which is more desirable than the white opaque appearance of micron sized metal oxide particles [1,6]. Besides, after reducing size to the nano-scale, the performance (UV attenuation) of these particles can be enhanced, which has been verified by both theoretical and experimental studies [7-9]. However, in spite of their ability to efficiently block UV radiation, concerns have been raised about the environmental impact and potential toxicity of these metal oxide nanoparticles [9,10].

It is well-known that the photocatalytic activity of metal oxide nanoparticles can result in free radical generation, which has been proven to damage DNA or tissues [1,9,11]. Although some metal oxide nanoparticles can be modified with non-semiconductor materials to reduce the generation of reactive oxygen species [1,12], other biosafety concerns, such as uptake and the interaction of nanoparticles with biological tissues still exist [12]. Though significant penetration of nanoparticles contained in sunscreens through the intact epidermal layer of skin has not been observed till now [1,13], human skin is not an impenetrable barrier. Hair follicles and abrasions provide opportunities for penetration into the vasculature [9,14]. A previous study indicated that 40 nm nanoparticles could penetrate through follicular openings and enter epidermal cells [15]. The increased use of synthetic nanoparticles has also been reported as a source of environmental contamination that may affect the ecosystem. Studies showed that TiO_2_ nanoparticles released into the aquatic environment may have long-term toxic effects due to their prolonged stability [16,17]. Besides, the toxic bioaccumulation caused by the transfer of nanomaterials among species raised more concerns [6,18]. Although many studies have been carried out to understand the scope and breadth of these potential hazards, a consensus about nanoparticle toxicity has not been reached [19,20].

Due to the potential hazards associated with the utilizing of metal oxide nanoparticles in sunscreen products, new green nanomaterials which are harmless to both human health and environment while providing similar UV protective effects are highly desirable. Naturally occurring ivy nanoparticles, which are secreted from the adventitious roots of English ivy (*Hedera helix*) [21], have been proposed for sunscreen applications [6,22]. Inherent properties of the natural organic nanoparticles usually endow them with less biosafety or environmental compatibility concerns compared to inorganic counterparts. As one special case of naturally occurring nanostructures [23], ivy nanoparticles are secreted from the root hairs accompanying with the secretion process of the ivy adhesive [24], forming a matrix with other components to support the surface climbing [21,25]. Both experimental and theoretical studies have shown that ivy nanoparticles have excellent transparency to visible light, and a strong ultraviolet extinction potential compared to TiO_2_ or ZnO nanoparticles [22]. Moreover, previous studies have indicated that ivy nanoparticles could be degraded by proteolytic enzymes, showed low cytotoxicity to mammalian cells, and had a limited possibility of penetrating human skin, all of which make ivy nanoparticles a promising candidate for sunscreen fillers [6].

However, before practical application of ivy nanoparticles to cosmetic fields, the physicochemical properties of these nanoparticles should be investigated. Nanoparticles behave differently from other micro- or macro-scale materials, due to the larger surface-to-volume ratio, which allows more atoms or molecules to be displayed on the surfaces [12]. The impact of particle size on the properties of materials can be illustrated by the case of TiO_2_ nanoparticles. TiO_2_ nanoparticles demonstrate rutile phase while particle size is above ~20 nm, whereas they exist in the form of anatase phase while particle size is below ~20 nm. [10,26]. Rutile TiO_2_ nanoparticles are usually used for sunscreen fillers, while anatase nanoparticles have been applied to self-cleaning glasses. The anatase-to-rutile phase transition is not only dependent on the particle size, but also related to other parameters, such as temperature [27], reaction atmosphere [28], and synthesis conditions [26,29]. Besides, the final nanomorphology of TiO_2_ depends upon the pH value, and hence its properties are sensitive to the resultant chemistry at the surface [26,30]. Therefore, a detailed understanding of the relationship between the function of nanoparticles and their physicochemical properties must be carried out before related products could be produced and commercialized [10]. Moreover, nanoparticles are also sensitive to the ambient environment. Environmental changes, such as temperature, pressure, or humidity, may alter the performance of nanomaterials [10]. For example, electromagnetic irradiation can permanently alter the shape of colloidal silver nanoparticles, thus affecting the surface plasmon resonances [31,32]. Due to the high surface-to-volume ratio of nanoparticles, it is challenging to maintain the stable surfaces of these nanomaterials both in device and in storage media. Changes of temperature or pH often influence the surface reactivity or desorb stabilizing surfactants on the surfaces, and hence cause the agglomeration of nanoparticles [10]. As naturally occurring nanoparticles, ivy nanoparticles may offer more complicated composition than synthetic inorganic nanoparticles. Thus, in-depth investigation on their physicochemical properties is necessary before practical applications. This study is not only necessary for the efficient use of ivy nanoparticles in the sunscreen industry but also for eliminating potential biosafety concerns about utilizing ivy nanoparticles.

In this study, the UV extinction properties of ivy nanoparticles were measured and analyzed under various temperatures and pH values. In addition, the influence of prolonged UV radiation on ivy nanoparticles was also investigated. Methylthiazol tetrazolium (MTT) assay was employed to study the cytocompatibility of this naturally occurring nanomaterial. Different from our early study, this research focused on the physicochemical properties analysis of ivy nanoparticles and data from this study provided a comprehensive understanding about the relationship between the UV extinction ability and the properties. Information gained through this study will advance potential applications of the ivy nanoparticles in sunscreen products.

## Materials and methods

### Materials

Juvenile shoots of English ivy were generously donated from Swan Valley Farms, Seattle. TiO_2_ and ZnO nanoparticles (diameter of 50 nm, 99% purity) were purchased from Nanostructured & Amorphous Materials Inc., Houston, TX. Murine melanoma B16BL6 cells, human non-small-cell lung cancer A549 cell lines were preserved by our own lab. Fetal bovine serum (FBS) and Dulbecco's modified Eagle's medium (DMEM) were purchased from Mediatech, Manassas, VA. Bovine serum albumin (BSA) was purchased from Sigma-Aldrich, St. Louis, MO. All other chemicals were purchased from Fisher Scientific, Pittsburgh, PA. All the water used for sample preparation and test was purified by a NANO pure Infinity-unit (Barnstead, Boston, MA).

### Ivy adventitious roots (rootlets) cultivation and nanoparticle isolation

Collected juvenile *Hedera helix* shoots were trimmed to 12 cm in length leaving only one piece of foliage on the head of each shoot fragment. The bottom of the resulting shoots were immersed into water for 24 hours, then incubated in 4.95 mM Indole-3-butyric acid Potassium (K-IBA) solution, and shaken at 100 rpm for 3 hours. After rinsing with water twice, shoots were then placed upright into Magenta GA7 boxes containing 50 ml water and were grown at 24°C at a 16:8 photoperiod under 82 μmol·m^-2^·s^-1^ irradiance. Ivy rootlets appeared at 8~10 days and grew to approximately 3 cm in length after one more week cultivation. Adventitious roots were then excised from each ivy shoot carefully to avoid potential physical damage to root hairs. Collected adventitious roots were then used immediately for isolation of ivy nanoparticles. After rinsing with distilled water twice, the wet ivy rootlets (10 g wet weight, from 200 ivy shoots) were extracted twice with 25 ml distilled water at 4°C in a sonicating bath for 1 hour while occasional mixing. The mixture was centrifuged at 12000 rpm for 1 min to remove chunks of debris and the supernatant was filtered through a 0.22 μm filter (Millex®GP, Millipore) to remove impurities. Large agglomerates of ivy nanoparticles were also removed from the resulting decentralized solution. The resulting filtrate was then dialyzed with a 300 kDa cut-off dialysis membrane (Spectra/Por® Biotech) against distilled water for three days with five changes to further purify the ivy nanoparticles and homogenize the size. Most of the small molecules such as soluble proteins, pigments and salts, were removed during dialysis. Isolated ivy nanoparticles were preserved in distilled water at 4°C for further study. Protein concentration of ivy nanoparticles was quantitatively determined by BCA protein assay kit (Thermo, IL).

### Influence of temperature variations on ivy nanoparticles

Freshly extracted ivy nanoparticles were diluted 6 times with distilled water to a final concentration of 500 μg/ml. 4 ml of diluted ivy nanoparticles were then treated at 7 different temperatures (-70°C, -20°C, 0°C, 20°C, 30°C, 40°C, 100°C) for two hours. After returning to room temperature (20°C), the size distribution, mean size and zeta potential of differently treated ivy nanoparticles were analyzed using dynamic light scattering (DLS, Zetasizer nano series, Malvern) measurements. The UV extinction spectra of differently treated ivy nanoparticles were measured using a UV-Vis spectrophotometer (Thermo Scientific Evolution 600 UV-Visible spectrophotometer, Thermo Fisher Scientific) from 260 nm to 400 nm. The optical length of the quartz cuvette was 1 cm. Detailed studies on the influence of an extreme temperature (100°C) on ivy nanoparticles were conducted using spectrofluorimetry (LS-50B, Perkin Elmer). Structural changes or unfolding of the ivy nanoparticles was monitored by changes of intrinsic protein fluorescence as described [33,34]. Emission spectra were recorded in triplicate between 260 and 420 nm using an excitation wavelength of 280 nm. The path length of the microvolume quartz cuvette was 1 cm. Emission spectra of ivy nanoparticles treated at other 6 temperatures (-70°C, -20°C, 0°C, 20°C, 30°C, 40°C) were recorded as the control.

### Influence of pH value changes on ivy nanoparticles

Freshly extracted ivy nanoparticles were diluted 6 times with distilled water and acetate buffer (0.1 M, pH 4.0) or phosphate buffer (0.1 M, pH 7.4) to a final concentration of 500 μg/ml. The pH value was adjusted during dilution. The final buffers used in this study were acetate buffer (0.02 M) with 3 different pH values (pH 4.0, pH 5.0 and pH 6.0) and phosphate buffer (0.02 M) with 4 different pH values (pH 7.4, pH 8.0, pH 9.0 and pH 10.0). Ivy nanoparticles dispersed in each buffer were incubated at room temperature for 2 hours followed by DLS measurements. The UV extinction spectra of the ivy nanoparticles at different pH values were measured as described earlier.

### Morphological study of ivy nanoparticles

To study the morphology of the ivy nanoparticles and the function of protein on maintaining this nano-morphology, atomic force microscopy (AFM) was performed using an Agilent 6000ILM/AFM (Agilent Technologies, CA). 10 μg of lyophilized ivy nanoparticles were dispersed in 400 μl distilled water and treated with an equal volume of phenol: chloroform: isoamyl alcohol (25: 24: 1, v/v/v). The mixture was stirred for 5 s and centrifuged at 12000 rpm for 10 min at room temperature. The aqueous phase was removed and stored at 4°C prior to analysis. 20 μl of the resulting ivy nanoparticles extract was deposited on a freshly cleaved mica substrate followed by air-drying overnight. Mica substrates were then stored in a desiccator at room temperature prior to AFM analysis. Samples were imaged at room temperature (20°C) using Picoview™ in AC mode. AFM probes were commercially available silicon probes PPP-NCHR-20 (Nanosensors™) with a force constant of 10-130 N/m and resonance frequency of 204-497 kHz. Scan rate was 0.5 ln/s. Un-treated ivy nanoparticles at the same concentration were scanned with the same AFM setting as the control.

### Influence of UV radiation on ivy nanoparticles

Freshly isolated ivy nanoparticles were diluted with distilled water 6 times to a final concentration of 500 μg/ml. Diluted samples were added to 12-well tissue culture polystyrene plates (TCPs, Becton Dickinson) at 2 ml/well. TCPs were allowed to air-dry overnight at room temperature. Each well of the TCPs was then covered by one coverslip. Subsequently, TCPs were placed into a biological safety cabinet (BSC, 1300 Series A2, Thermo Scientific) and irradiated with ultraviolet light followed by removing one column of coverslips at a fixed time (0 h, 8 h, 11 h). The UV source was a USHIO G36T5L 39-W/UV-C quartz lamp emitting radiation peaking at 253.7 nm. The distance between the TCPs and the ultraviolet lamp was 75 cm. After 12 h irradiation, ivy nanoparticle precipitates in each well were re-suspended and dispersed with 2 ml of distilled water followed by UV-Vis measurements to record the UV extinction spectra of the ivy nanoparticles under different ultraviolet exposure times. The BSA with the same concentration was treated based on the same procedure as the control.

### Visual transparency analysis and MTT assay

Lyophilized ivy nanoparticles were dispersed with distilled water at the concentration of 500 μg/ml, 100 μg/ml and 10 μg/ml. The transparency of dispersed ivy nanoparticles was visually compared to TiO_2_ and ZnO nanoparticles with the same concentration. MTT assay was also performed to compare the cytotoxicity of ivy nanoparticles with TiO_2_ and ZnO nanoparticles. A549 and B16BL6 tumor cells were cultured in a DMEM with 10% FBS and an atmosphere of 5% CO_2_ in air at 37°C. After incubating with serial diluted nanoparticles for 48 hours, MTT assay was conducted and absorbance at 570 nm was recorded.

### Statistical analysis

All quantitative results are representative of three independent experiments. Data were expressed as the mean ± standard deviation (SD). Statistical analysis was performed using one-way ANOVA, performed with a computer statistical program (PASW Statistics 18). A value of P < 0.05 was considered to be statistically significant. Tukey post hoc multiple comparisons were performed to determine differences between every two groups.

## Results and discussion

### Ivy nanoparticle isolation and purification

**Figure 1:**
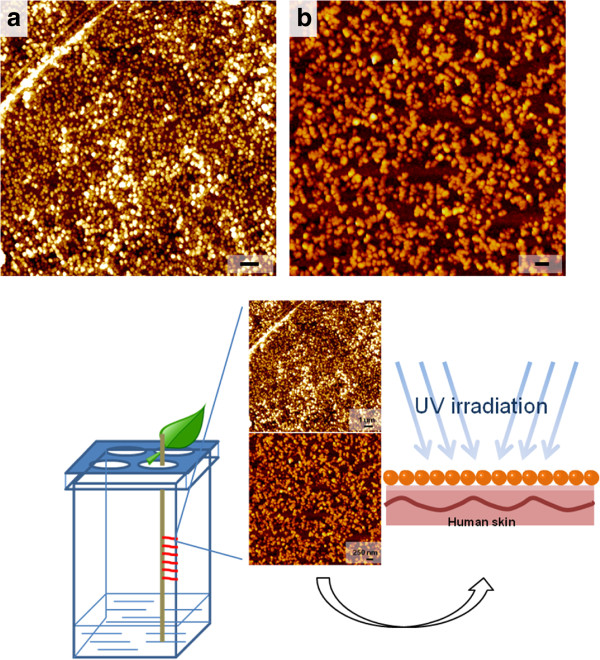
**AFM images of purified ivy nanoparticles and schema of UV protection provided by ivy nanoparticles.** The scale bars represent 1 μm in image **a**, and 250 nm in image **b.**

In order to facilitate large-scale applications of ivy nanoparticles in cosmetic products, a convenient and reliable system for ivy cultivation, nanoparticle isolation and purification was developed. Different from common tissue culture methods used before [6], this improved cultivation system avoids complicated sterilizing procedures, but uses water alone as the growth source instead of an organic culture medium. Using this controllable cultivation system, contamination and environmental influence was minimized. DLS and AFM studies verified the secretion of ivy nanoparticles from ivy rootlets cultivated by this system [6,21]. Figure 1 shows the result of the purified ivy nanoparticles. After isolation and purification, ivy nanoparticles were distributed on the mica surface. The size of ivy nanoparticles was around 80 to 140 nm, which was consistent with previous reports [6]. Isolated ivy nanoparticles were preserved in distilled water at 4°C for further study. Freshly isolated nanoparticles were tested or studied no more than 3 days after isolation.

### Temperature effect

**Figure 2:**
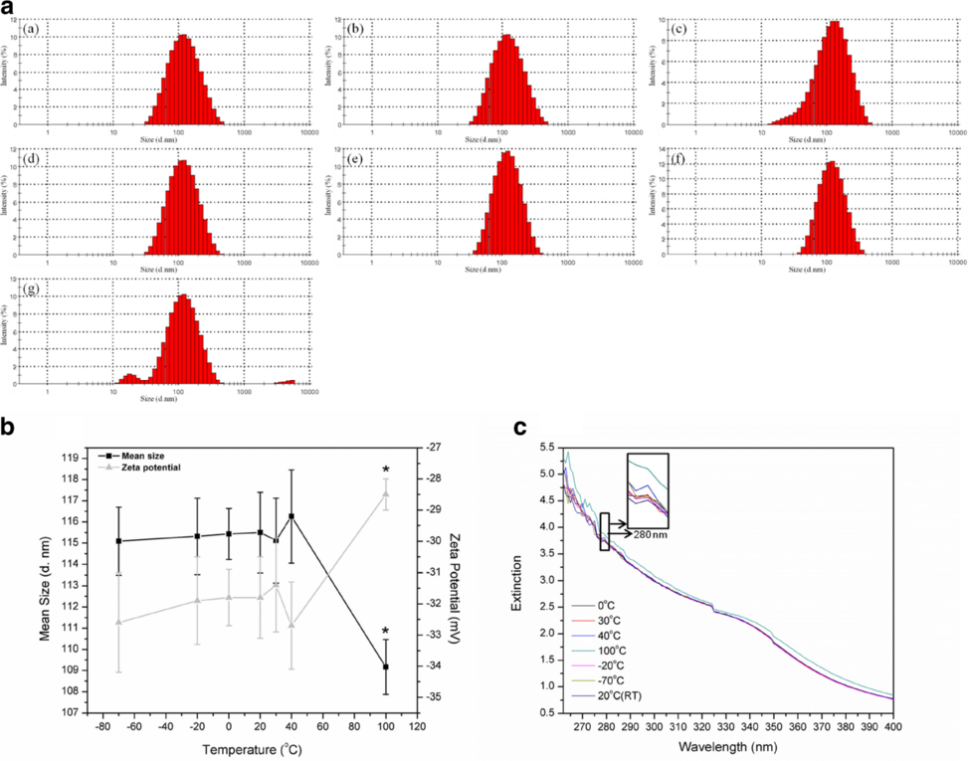
**Temperature influence on ivy nanoparticles. a** Size distributions of ivy nanoparticles treated with different temperatures: (a) -70°C, (b) -20°C, (c) 0°C, (d) 20°C, (e) 30°C, (f) 40°C, (g) 100°C, **b** Mean sizes and zeta potentials of ivy nanoparticles treated with 7 different temperatures, and **c** UV extinction spectra of ivy nanoparticles treated with 7 different temperatures. The panel is the amplification of the corresponding area at 280 nm.

Previous studies indicated that ivy nanoparticles show increased extinction spectra in the UVA/UVB range and decreased absorbance in the visible region when compared to TiO_2_ nanoparticles, which endow ivy nanoparticles with a greater UV protective potential and allow them to be transparent to visible light. In the meantime, previous report also mentioned the possibility of investigating the UV extinction property of ivy nanoparticles from a protein perspective [6]. By BCA quantification, the concentration of protein in ivy nanoparticles was around 58%. In this study, factors which may have a possible impact on the morphology and surface reactivity of ivy nanoparticles, especially the influence on protein stabilities, were investigated. The influence of temperature variation on the morphology of ivy nanoparticles and on the UV extinction capability was evaluated by DLS and UV-Vis spectrophotometry. Freshly isolated ivy nanoparticles were treated at 7 different temperatures for 2 hours followed by DLS measurements to obtain the particle size distribution, mean size and zeta potential variations [35,36]. The mean from three separate trials was compared to determine the standard deviation from experiment to experiment. Results from the measurement show that the mean size of the extracted ivy nanoparticles preserved at room temperature was 115.50 ±1.9 nm (SD stands for the standard deviation of three mean sizes getting from three independent trials, Figure 2b), while 90.3% of the nanoparticles were between 70.90 to 255 nm (Figure 2a). The lowest mean size (109.17 ±1.3 nm) was obtained by the ivy nanoparticles treated at 100°C, whereas ivy nanoparticles treated with other temperatures showed little difference in the mean size (Figure 2b). After treating at 100°C for 2 hours, 1.1% of ivy nanoparticles were agglomerated to larger than 4 μm, and 5.2% were in the form of small (11.61~32 nm) nanostructures (Figure 2a). As shown in Figure 2a, ivy nanoparticles demonstrated great temperature tolerance over a wide range of variation. Even treated with the extreme temperature for a long time, most ivy nanoparticles still maintained stable nanostructures and steady dispersive state. The temperature-tolerant behavior of ivy nanoparticles was consistent with the physiological characteristics of this plant, which can survive in a wide range of ambient temperatures. Ivy nanoparticles were indicated to participate in the climbing process [21,24,25], in which tolerance over a broad temperature range is an important feature. Zeta potential analysis revealed that the ivy nanoparticles treated at 100°C demonstrated a slightly increased zeta potential compared to other samples, which suggested an increasingly unstable dispersive state of nanoparticles at this temperature (Figure 2b).

Subsequently, the influence of temperature on the UV extinction spectra was further evaluated by UV-Vis spectrophotometry. 3 ml aqueous solutions of ivy nanoparticles treated at different temperatures were added to quartz cuvettes, and the absorbance was recorded. Figure 2c shows that the ivy nanoparticles demonstrated strong extinction spectra from 260 to 400 nm (UVA/UVB range), which was consistent with previous reports [6]. However, the UVA/UVB extinction spectra of ivy nanoparticles treated at 100°C displayed a distinct increase compared to other samples. Compared to the results obtained from DLS, this increased absorbance was attributed to the 5.2% small nano-groups (11.61-32 nm) caused by partial decomposition of the ivy nanoparticles after treating at 100°C. The increased surface-to-volume ratio is believed to enhance the UV absorption of this sample.

**Figure 3:**
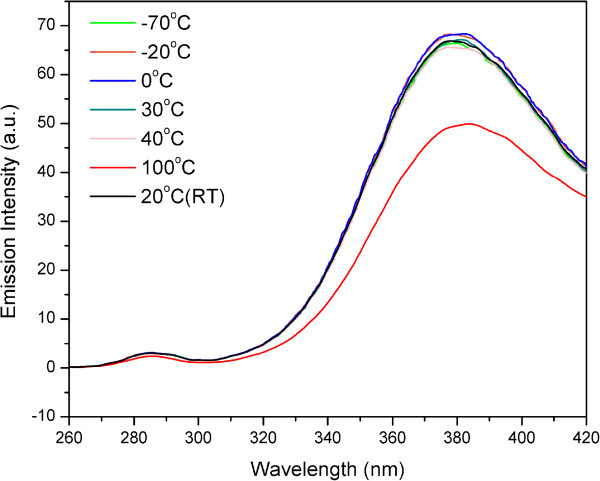
**Intrinsic fluorescence of protein in ivy nanoparticles.** Emission spectra of ivy nanoparticles with an excitation of 280 nm. Ivy nanoparticles were treated under -70°C, -20°C, 0°C, 20°C, 30°C, 40°C and 100°C respectively.

The structural changes of ivy nanoparticles treated at 100°C were examined by spectrofluorimetry. Intrinsic fluorescence of protein was usually used to observe the denaturation or unfolding of macromolecules [34,37,38]. Fluorescence measurements monitor the state of aromatic side chains within the protein (usually tryptophan due to its strong quantum yield), and intrinsic protein fluorescence can be measured at approximately 350 nm after exciting with 280 nm ultraviolet light while the actual emission wavelength can vary depending on the polarity of the environment. Comparing to the folded state, the quantum yield may be either increased or decreased by the unfolding because the fluorescence of the aromatic residues varies in somewhat unpredictable manner in various proteins. Accordingly, an unfolded protein can have either greater or less fluorescence than the folded form [39]. In this study, aqueous solution of ivy nanoparticles treated at 100°C was excited at 280 nm and emission spectra were recorded from 260 to 420 nm. As shown in Figure 3, ivy nanoparticles preserved at room temperature demonstrated an emission wavelength of around 375 nm. This small drift from the theoretical value (350 nm) was attributed to the interference of other chemical components in ivy nanoparticles. The emission spectra noticeably decreased after treating at 100°C, which indicated the partial denaturation of proteins occurring in ivy nanoparticles. Comparatively, ivy nanoparticles treated with other temperatures didn’t show any significant difference in the emission spectra with each other. Together with results from DLS measurements, it was evident that the degradation and agglomeration process of ivy nanoparticles were accompanied by the partial unfolding process of proteins, which implied that proteins played an important role in maintaining the three-dimensional structures of ivy nanoparticles.

### Stability of ivy nanoparticles to pH

**Figure 4:**
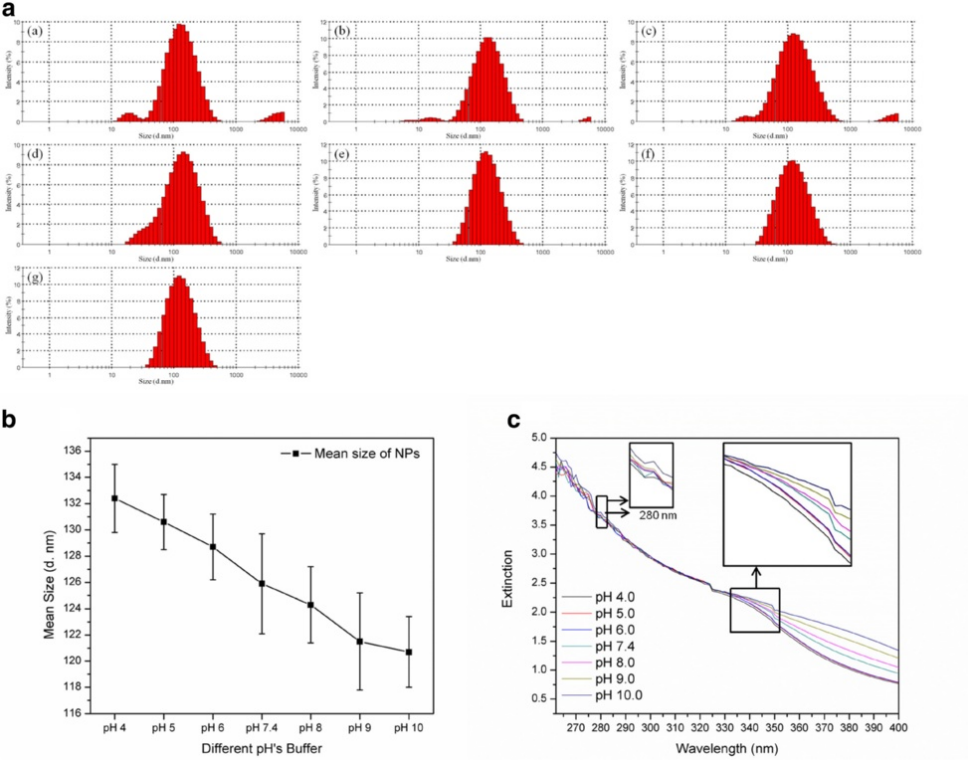
**The influence of pH on ivy nanoparticles. a** Size distributions of ivy nanoparticles dispersed in buffers with different pH values: (a) pH 4.0, (b) pH 5.0 (c) pH 6.0, (d) pH 7.4, (e) pH 8.0, (f) pH 9.0, (e) pH 10.0, **b** Mean sizes of ivy nanoparticles dispersed in buffers with 7 different pH values, and **c** UV extinction spectra of ivy nanoparticles dispersed in buffers with 7 different pH values. The panels are the amplification of the corresponding areas of the UV extinction spectra.

The sensitivity to pH value was also an important factor when evaluating the application of ivy nanoparticles in sunscreen products [40]. Investigation of the influence of pH variation on the stability and agglomeration of ivy nanoparticles was not only useful for manipulation but also valuable for practical utilizing. In this study, ivy nanoparticles were dispersed in buffers with 7 different pH values followed by DLS measurements and UV-Vis analysis. Results show that ivy nanoparticles demonstrated diverse mean sizes after dispersing in buffers with different pH values (Figure 4b). The mean size increased from 120.7 ±2.7 to 132.4 ±2.6 nm (SD stands for the standard deviation of three mean sizes getting from three independent trials) accompanying with the decrease of pH values (Figure 4b), which implied that ivy nanoparticles were more stably dispersed in alkaline environments than in acidic ones. Ivy nanoparticles showed more degradation and agglomeration while dispersing in acidic environments (pH 4.0, pH 5.0 and pH 6.0) (Figure 4a). For example, 3.2% of ivy nanoparticles were in very small sizes (17.6~22.5 nm) and 3.9% were agglomerated to larger than 4 μm while dispersing in acetate buffer (0.02 M, pH 4.0) (Figure 4a). These results collectively suggested that ivy nanoparticles were more sensitive to acidic solutions than alkaline ones. As discussed before, protein played an important role in adjusting three-dimensional structures of ivy nanoparticles, thus should be sensitive to pH variations. It was also noticed that the mean size for ivy nanoparticles dispersed in buffers was larger than nanoparticles dispersed in distilled water (Figure 2b, 4b). This difference was attributed to the interference of salt ions.

The UV extinction spectra of ivy nanoparticles dispersed in buffers at different pH’s were also recorded using UV-Vis spectrophotometry. As shown in Figure 4c, the UV extinction spectra were measured from 260 to 400 nm. Within the wavelength between 260 and 320 nm (UVB), no distinct spectral difference was observed among different pH values, whereas the UV extinction spectra from 320 to 400 nm (UVA) decreased significantly accompanying with the decrease of pH values. This sub-regional extinction decrease may be attributed to agglomeration of the ivy nanoparticles (Figure 4b). Reduced surface-to-volume ratio may lower the absorption of ultraviolet light. However, the difference between UVA and UVB regions needs to be further investigated. Also, this sub-regional absorbance difference provided a unique channel to utilize ivy nanoparticles in sunscreen products.

### Morphological study

**Figure 5:**
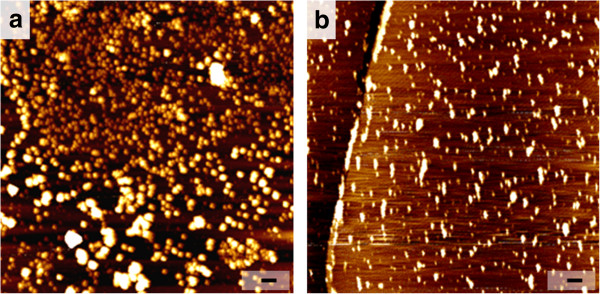
**AFM study on the morphology of ivy nanoparticles. a** AFM image of ivy nanoparticles on the surface of mica, and **b** AFM image of ivy nanoparticles’ residues after extracting with phenol-chloroform-isoamyl alcohol. The scale bar represents 250 nm for both images.

Previous AFM studies indicated that the morphology of ivy nanoparticles was stable but could be digested by Proteinase K [6]. Since proteins may play an important role in modulating the three-dimensional structures of the ivy nanoparticles, a harsh treatment (phenol-chloroform extraction) was employed to eliminate most of the proteins from the nanoparticle structures followed by AFM analysis. Un-treated ivy nanoparticles at the same concentration were scanned with AFM as a control. As shown in Figure 5, natural ivy nanoparticles displayed spherical ordered structure and were densely distributed on the mica surface. The morphology was similar to previous reports [21,25]. However, after applying phenol-chloroform-isoamyl alcohol, the three-dimensional structure of ivy nanoparticles was distinctly affected. Small debris appeared on the surface of mica instead of uniform nanoparticles and was displayed by irregular forms. The size of these residues was much smaller than that of intact nanoparticles. Remaining components of the debris on the mica substrate were speculated to be polysaccharides and/or plant secondary metabolites, which needed further chemical analysis. This morphological change verified the protein as a necessary component to maintain the structure of ivy nanoparticles.

### UV irradiation influence

**Figure 6:**
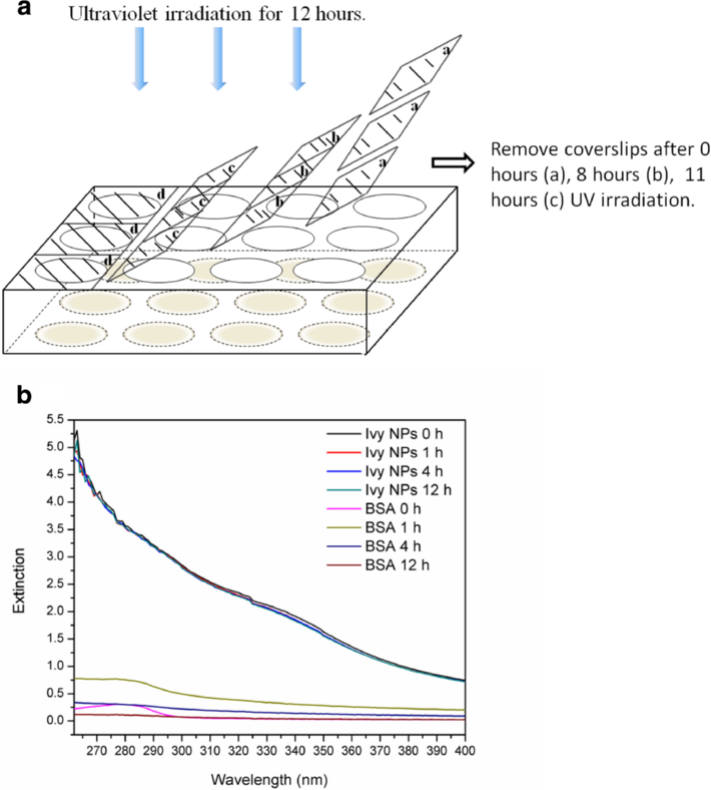
**UV irradiation influence on ivy nanoparticles. a** Schema of UV irradiation test. Irradiation time was controlled by removing coverslips at scheduled time (0 hour, 8 hours and 11hours), and **b** UV spectra of ivy nanoparticles after treatment with different UV irradiation time. The BSA with the same concentration was treated based on the same procedure as the control.

Since ivy nanoparticles were proposed to be a suitable substitute for metal oxide nanoparticles in sunscreen products [6,22], more detailed studies on the influence of UV irradiation on nanoparticle itself became particularly important. It was reported that after irradiation with a conventional 40-W fluorescent light for 70 hours, silver nanospheres showed a concomitant growth of three new bands and a decrease of the characteristic band at 400 nm in the UV-Vis spectroscopy [31]. Since ivy nanoparticles are regulated by their protein structure, they may be more sensitive to UV-irradiation as proteins are usually more fragile to UV irradiation. Oxidative damage to the collagen protein caused by UV radiation has also been widely studied [41-43]. The UV radiation could reduce fibrillogenesis level while cleaving peptide bonds of collagen randomly. In our study, ivy nanoparticles were air-dried in the wells of TCPs followed by UV irradiation with 4 different time scales (0 hour, 1 hour, 4 hours, 12 hours). A 39-W UV light source from a BSC, which emitted radiation peaking at 253.7 nm was used as the UV radiation source. The irradiation time was well controlled by removing the covered glass sheets at scheduled times (Figure 6a) since all UV light below 300 nm could be blocked by the cover glass [44-46]. The BSA was utilized as the control. After irradiation, ivy nanoparticles treated with different radiation durations were re-suspended and dispersed with distilled water and the UV extinction spectra were recorded to evaluate the influence of UV irradiation on nanoparticles. Results show that the extinction spectra of ivy nanoparticles displayed a slight decrease from a wavelength of 350 to 400 nm after treatment with UV irradiation, but this impact was time-independent (Figure 6b). However, the UV extinction spectra of the BSA demonstrated a clear variation with the change of UV irradiation time. Untreated BSA showed a characteristic band at 280 nm. After UV irradiation for 1 hour, the overall UV extinction spectra increased; however, the band at 280 nm became broader. With an increasing duration of UV irradiation, the UV extinction spectra gradually decreased and almost disappeared at 12 hours. We believed that this variation was attributed to the intramolecular disordered movement and the breaking of aromatic groups caused by the consistent absorbing energy under UV radiation. This study indicated that ivy nanoparticles were relatively stable and reliable to serve as a UV filter substrate.

### Nanoparticles transparency analysis and cytocompatibility

**Figure 7:**
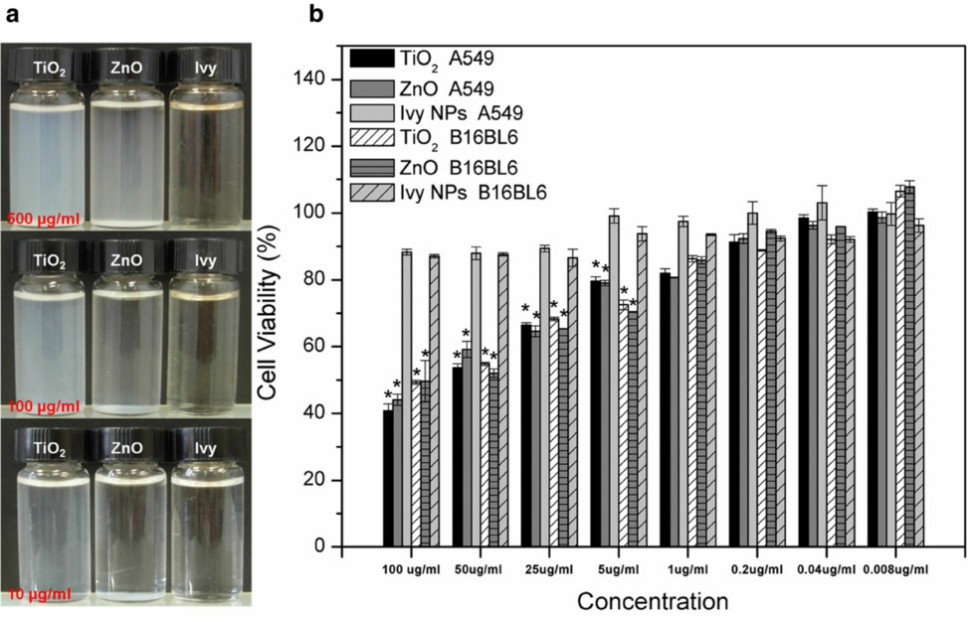
**Visual transparency analysis and cytocompatibility study. a** Visual transparency comparison of three different nanoparticles (ivy, TiO_2_, ZnO) dispersed in distilled water with three different concentrations (500 μg/ml, 100 μg/ml and 10 μg/ml), and **b** MTT assay to compare the cytotoxicity of three different nanoparticles (ivy, TiO_2_, ZnO). Nanoparticles were gradiently diluted to 100 μg/ml, 50 μg/ml, 25 μg/ml, 5 μg/ml, 1 μg/ml, 0.2 μg/ml, 0.04 μg/ml and 0.008 μg/ml.

Visual transparency is a vital merit for nanoparticles to be used as sunscreen fillers. In this study, the visually transparent property of ivy nanoparticles was compared to TiO_2_ and ZnO nanoparticles after dispersing in distilled water. Results indicated that ivy nanoparticles presented better transparency in liquid solution (Figure 7a). MTT assay was also performed to evaluate the cytotoxicity of three different nanoparticles. Ivy nanoparticles showed lower cytotoxicity to A549 and B16BL6 tumor cells compared to TiO_2_ and ZnO nanoparticles (Figure 7b). For concentrations above 5 μg/ml, metal oxide nanoparticles showed distinct cytotoxicity, whereas ivy nanoparticles had little effect. Better cytocompatibility suggested the inherent advantages of naturally occurring nanomaterial.

## Conclusions

Ivy nanoparticles have been proposed as a potential substitute for metal oxide nanoparticles in sunscreens due to their effective UV extinction potential, low toxicity, and biodegradability [6]. In this study, the stability of the ivy nanoparticles, specifically their UV protective capabilities, to changes in temperature, pH, and prolonged UV exposure were investigated. Results showed that ivy nanoparticles demonstrated relatively strong temperature, pH and UV irradiation tolerance. However, at 100°C, ivy nanoparticles were partially degraded and displayed increased agglomeration, which were modulated by partial protein unfolding. This degradation led to an increase in the UV extinction spectra of ivy nanoparticles. Considering that 100°C falls well outside of the range necessary for a sunscreen product, the stability analysis from -20°C to 40°C shows that the ivy nanoparticles meet the criteria necessary for stable sunscreen filler. Since high temperatures may still be encountered in the process of manufacturing a sunscreen product incorporating the ivy nanoparticles, such as the sterilization of the ivy nanoparticles using autoclaving, this study had certain guiding significance to the practical operation. Results about the influence of pH variation on ivy nanoparticles showed that the UVA extinction spectra slightly decreased with a decrease of pH values. Nanoparticles were more stable in alkaline solutions than in acidic environments. It was also observed that protein played an important role in modulating three-dimensional structures of ivy nanoparticles. The morphology study showed that after removing most protein from ivy nanoparticles, there were still some small residues which displayed irregular and asymmetric structures on the surface of mica. Furthermore, the influence of UV irradiation on the ivy nanoparticles was evaluated by UV-Vis spectroscopy and the results indicated that the impact was small and time-independent. In summary, ivy nanoparticles demonstrate the necessary stability to be used as sunscreen filler, with advantages over currently used metal oxide nanoparticles. The increased visual transparency and safety of these nanoparticles make them an attractive candidate to replace metal oxide nanoparticles, leading to less concern over the environmental impact of these nanomaterials. Future studies will focus on isolation and identification proteins from ivy nanoparticles. The practical application of ivy nanoparticles for UV protection will also be evaluated.

## Competing interests

The authors declare that they have no competing interests.

## Authors’ contributions

YH performed the majority of the experiments, performed data analysis, performed statistical analysis and wrote the manuscript with SCL, LX and MZ. JNB designed the study on ivy cultivation. CNSJ co-designed the study, reviewed and revised the manuscript. All authors read and approved the manuscript.
